# Cabrol procedure and its modifications: a systematic review and meta-analysis

**DOI:** 10.1186/s13019-024-02642-w

**Published:** 2024-03-26

**Authors:** Sen Yang, Ya-yong Zhang, Yun-feng Zi, Lei Pu, Xu Qian, Le Ren, Yong-bo Li, Zhi-hao Jin, Jian-feng Liu, Zhuo Yuan, Ya-Xiong Li

**Affiliations:** 1 Department of Cardiovascular Surgery, Yan’an Affiliated Hospital of Kunming Medical University, Kunming, Yunnan China; 2 Key Laboratory of Cardiovascular Disease of Yunnan Province, Yan’an Affiliated Hospital of Kunming Medical University, Kunming, Yunnan China

**Keywords:** Cabrol procedure, Modified Cabrol procedures, Cabrol-related coronary graft complications

## Abstract

**Background:**

The Cabrol procedure has undergone various modifications and developments since its invention. However, there is a notable gap in the literature regarding meta-analyses assessing it.

**Methods:**

A systematic review and meta-analysis was conducted to evaluate the effectiveness and long-term outcomes of the Cabrol procedure and its modifications. Pooling was conducted using random effects model. Outcome events were reported as linearized occurrence rates (percentage per patient-year) with 95% confidence intervals.

**Results:**

A total of 14 studies involving 833 patients (mean age: 50.8 years; 68.0% male) were included in this meta-analysis. The pooled all-cause early mortality was 9.0% (66 patients), and the combined rate of reoperation due to bleeding was 4.9% (17 patients). During the average 4.4-year follow-up (3,727.3 patient-years), the annual occurrence rates (linearized) for complications were as follows: 3.63% (2.79–4.73) for late mortality, 0.64% (0.35–1.16) for aortic root reoperation, 0.57% (0.25–1.31) for hemorrhage events, 0.66% (0.16–2.74) for thromboembolism, 0.60% (0.29–1.26) for endocarditis, 2.32% (1.04–5.16) for major valve-related adverse events, and 0.58% (0.34–1.00) for Cabrol-related coronary graft complications.

**Conclusion:**

This systematic review provides evidence that the outcomes of the Cabrol procedure and its modifications are acceptable in terms of mortality, reoperation, anticoagulation, and valve-related complications, especially in Cabrol-related coronary graft complications. Notably, the majority of Cabrol procedures were performed in reoperations and complex cases. Furthermore, the design and anastomosis of the Dacron interposition graft for coronary reimplantation, considering natural anatomy and physiological hemodynamics, may promise future advancements in this field.

**Supplementary Information:**

The online version contains supplementary material available at 10.1186/s13019-024-02642-w.

## Background

The development and the increasing utilization of valve-sparing aortic root replacement (VSRR), which preserves the native valve and carries a lower risk of hemorrhage and thrombosis, has established it as the primary treatment option for aortic root aneurysms, especially in younger patients. However, composite valve graft (CVG) continue to be the predominant approach in managing aortic root diseases, offering significant advantages such as long-term valve durability, operational stability, and the ability to be implemented across multiple medical centers [1]. Research conducted by Stamou et al. [2] revealed that the proportion of patients undergoing VSRR remains below 15% in the United States. Furthermore, based on the STS adult cardiac surgery database, Wallen et al. [3] showed that CVG was used in 81% of aortic root replacement procedures between 2011 and 2016, while VSRR was used in the remaining cases.

The Cabrol procedure is an example of the use of CVG in aortic surgery, which was introduced by Cabrol and colleagues in 1981 [4], offering a tension-free anastomosis as an innovative alternative to the original Bentall procedure [5, 6]. The procedure attaches the aortic graft to the coronary ostia with a separate Dacron graft to prevent pseudoaneurysms due to excessive tension on the anastomosis [7, 8]. However, reports about stenosis, thrombosis, and occlusion of Dacron grafts have hindered the appilication of Cabrol procedure [7, 9–14]. As a result, some researchers have proposed their own improvements to the design and placement of the interposition graft to address the risks associated with twisting and angulation. Pierhler et al. [15] recommended that the left coronary ostia should be anastomosed to the composite conduit with an interposition graft, while the right coronary ostia were anastomosed directly to the composite conduit for simplifying the movement. Mills et al. [16] proposed the “leg” technique, in which short separate grafts were implanted from each coronary ostia into the composite valve graft. Different centers reported different opinions on the optimal length of the branch leg. For example, Maureira et al. [17] advocated using two separate 4–10 mm grafts for coronary artery reimplantation as a simple, reproducible, and safe technique. Our center favors a 3-4 mm interposition graft for enhanced effectiveness in minimizing complications and optimizing blood flow dynamics in the coronary arteries [18]. Kourliouros et al. [19] introduced the “T-fashion” modification. Meanwhile, the Cabrol procedure and its modifications have been tested and confirmed to be safe and effective [17, 20–23]. It’s currently indicated when traditional button implantation is difficult, such as fragile or torn coronary ostia. Other indications include reoperation, low coronary ostia (< 1.5 cm above the aortic valve annulus), aortic calcification and severe dissection, it is usually applied for unforeseen complications in routine aortic surgery [10, 12, 20, 24–26].

Surprisingly, there is a scarcity of systematic reviews and meta-analyses investigating the outcomes and long-term prognosis of the Cabrol procedure in the existing literature, except the Cabrol-related review published by Kourliouros et al. [26] in 2011. To address this gap and to comprehensively evaluate the strengths and weaknesses of the Cabrol procedure and its modifications, a systematic review and meta-analysis was conducted, focusing on the perioperative characteristics and long-term outcomes. This study serves as a valuable resource for surgeons and medical centers, providing a reference to assess the potential effectiveness of new techniques and guide the selection of safer and more appropriate procedures.

## Materials and methods

### Search strategy

This meta-analysis adheres to the guidelines outlined by the Preferred Reporting Items for Systematic Reviews and Meta-Analysis (PRISMA), a program registered in the International Prospective Register of Systematic Reviews (PROSPERO identifier: CRD42023430388). Since all analyses were conducted using previously published studies, the need for ethical approval and patient consent were not required.

A comprehensive strategy was utilized by 2 researchers (Z.Y.F and P.L) working independently and to search in the Pubmed, Embase and Web of Science from inception to April 2023, identifying the relevant studies using the following combination of subject terms and free text terms: “Cabrol”, “modified cabrol”, “aortic root replacement”, “composite valve graft”, “aortic root aneurysm”, “ascending aortic aneurysm”, “dissection, ascending aorta” and “aortic valve insufficiency”, more specific details were provided in the supplementary materials (Supplementary Appendix 1).

### Inclusion and exclusion criteria

Only studies reporting outcomes of 15 or more patients aged 18 years or older who underwent the Cabrol procedure and its modifications were included. When evaluating a larger series of different aortic root procedures, the specific outcomes related to the Cabrol subgroup were examined, which included morbidity, mortality and coronary graft complications associated with the Cabrol technique. In cases where multiple publications existed for a single study, the most recent and comprehensive data were selected, and all selected studies were cross-referenced to identify additional relevant publications. Only full articles written in English were included, and in cases where the reviewers disagreed about the inclusion of a publication, consensus was reached.

### Data extraction

Three authors (Q.X, R.L and L.Y.B) independently extracted data using Microsoft Excel (Microsoft Office 2021, Microsoft Corp, Redmond, WA) in accordance with the guidelines for reporting mortality and morbidity after cardiac valve interventions [27]. The relevant data were extracted from the reviewed text, tables, and graphs of the papers. The collected data encompassed all relevant variables pertaining to the patients’ preoperative, postoperative, and follow-up periods. Events that did not comply with reporting guidelines were excluded from our database. For articles lacking information on important variables, the corresponding authors were contacted to provide the missing data. Any disputes arising during the data extraction process were resolved through collaborative negotiation and consensus among the three investigators. A comprehensive overview of the extracted variables is provided in the supplementary materials (Supplementary Appendix 2).

### Data synthesis and statistical analysis

During the evaluation process, the extracted data were analyzed using Microsoft Excel (Microsoft Office 2021, Microsoft Corp, Redmond, WA) and Stata version 15.0 (Stata Corporation, College Station, TX). The reported characteristics of the included studies were presented as means and standard deviations for continuous variables, and percentages for discrete variables. To investigate the correlation between the surgical period and outcomes following the Cabrol procedures, we defined the continuous variable “surgical period” as the year when the first patient was included in each cohort. The outcome events were reported as linearized incidence rates, expressed as percentages per patient-year. The number of patient-years was calculated by multiplying the number of patients included in the study by the mean follow-up time (in years), and the incidence per case was calculated by dividing the number of events by the total number of patient-years of follow-up. When a particular event did not occur in an individual study, we set the number of events to 0.5 in order to pool the linearised incidence of that particular event into the study. I^2^ statistics evaluated by the Q test were used to quantify the degree of heterogeneity between studies. Considering the inherent variation in study design, all values were calculated by using a random effects model [28]. Heterogeneity was analysed for outcomes with I^2^ > 50% [29].

To assess the relationship between six baseline variables (age, surgical period, proportion of patients with Marfan’s disease, type A aortic dissection and reoperation, and classical or modified Cabrol procedure) and nine significant outcome events (early mortality, bleeding reoperation, late mortality, root reoperation, hemorrhage, embolism, endocarditis, major valve-related adverse events, and coronary graft complications) relationships, linear regression analyses were performed, and regression analyses were weighted by study size using the inverse variance method. Sources of heterogeneity were further discussed using sensitivity analysis. Finally, visual observation of Begg’s funnel chart along with Begg’s and Egger’s tests [30, 31] were used to assess publication bias, and a *P*-value of < 0.05 was considered statistically significant.

## Results

**Fig. 1:**
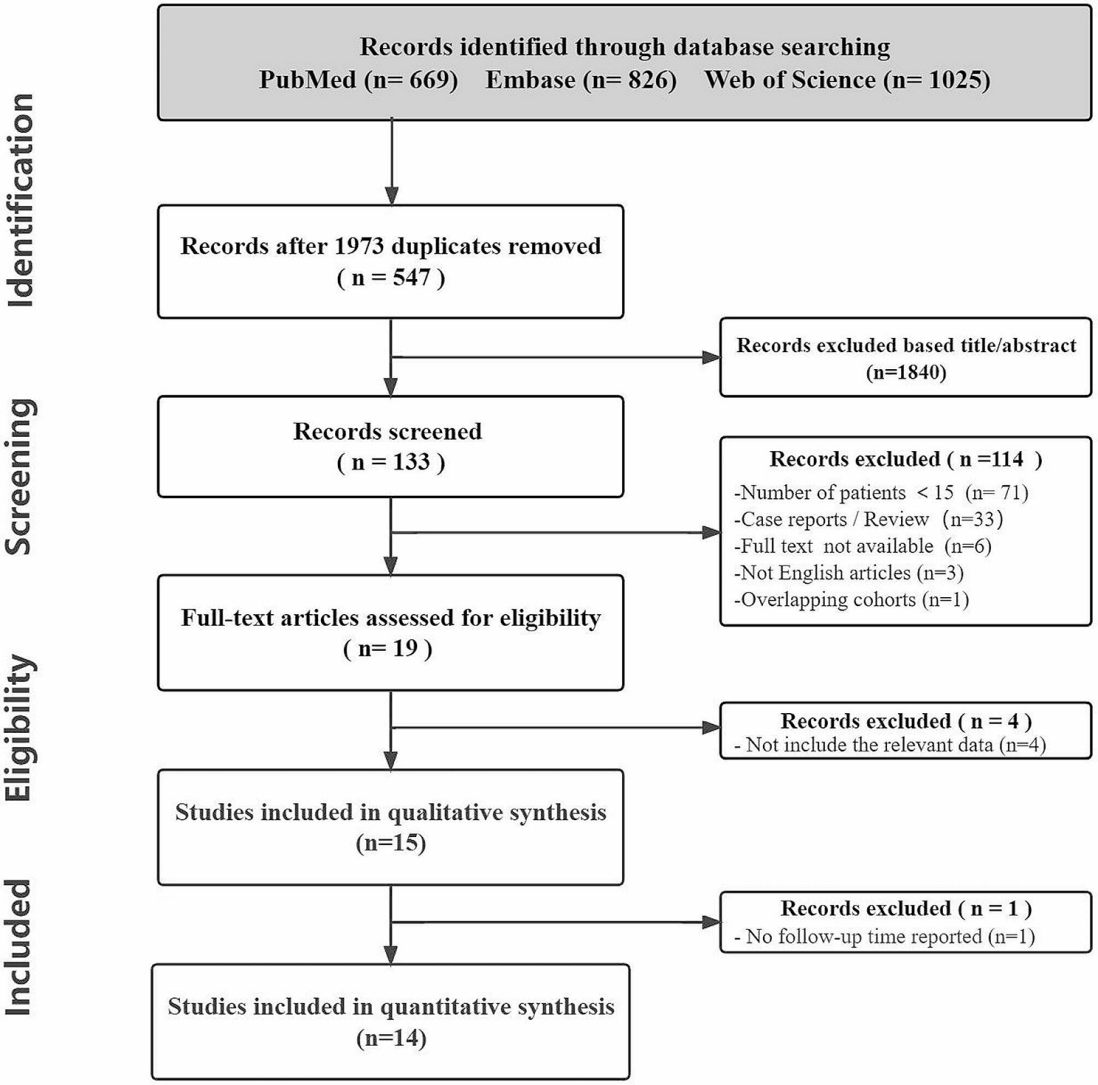
Flow chart of the selection process for studies included in the systematic review and meta-analysis

The comprehensive search yielded 2520 articles, of which 19 articles were potentially eligible after excluding duplicates and irrelevant articles by reading titles and abstracts. After full-text review, four articles were excluded because they did not provide data on Cabrol-related morbidity, mortality or graft complications. In addition, a study by Coselli et al. [32] was excluded from the quantitative analysis due to the lack of a description of the duration of follow-up. Therefore, 14 studies with a total of 833 patients were included in this meta-analysis. (Fig. 1) illustrates the selection process of the 14 articles. The pooled preoperative and perioperative characteristics are shown in (Table 1). The mean follow-up time after Cabrol surgery was 4.4 years (range 1.7–8.6 years) for a total of 3727.3 patient-years. The characteristics of the included studies are summarized in (Supplementary Appendix 3) [7–10, 12, 20–25, 33–35]. Consistently, classical Cabrol technique is still used in the majority of studies, with 80.7% (672/833) of the total patients in 11 studies using it [7–10, 12, 20, 21, 24, 25, 33, 34]. The proportion of reoperation was 34.5% (126/365) in the 8 studies [9, 12, 20–23, 33, 35]. 27.7% (96/347) of connective tissue disease in 7studies [9, 12, 20–23, 33]. Aortic dissection in 6 studies was 42.2% (136/322) [9, 12, 21–23, 33].

**Table 1:** Pooled preoperative and perioperative characteristics

Variables	Pooled Data	Data Range	Included Studies (N)
Total patient number	833	15–260	14
Surgical period	1973–2020		14
Mean age, y	50.8	42.5–60	12
Sex (%)			
Male	68.0	13–84	7
Comorbidity (%)			
Connective tissue disease Bicuspid aortic valve Coronary heart disease Hypertension Annuloaortic ectasia Atherosclerotic aneurysm Poststenotic dilatation	27.7 16.4 20.9 45.5 55.2 12.9 9.4	2–47 3–8 4–23 11–22 10–68 3–5 3–5	7 3 2 3 3 2 2
Previous operation (%)	34.5	3–62	8
Type A dissection (%) Acute	42.2 14.0	8–56 5–12	6 6
Endocarditis (%)	13.0	1–12	3
Emergency operation (%)	28.0	1–21	5
Surgical data			
Cardiopulmonary bypass time Aortic cross-clamp time	192.7 137.5	126–247 91–186	5 5
Cabrol types (%)			
Classic Modified Cabrol fifistula Mechanical prosthesis Bio-prosthesis	80.7 19.3 64.0 85.2 11.3	6-212 18–84 0-212 25–69 0–13	14 14 7 5 5
Concomitant procedures (%)			
Aortic arch repair CABG Mitral valve operation	18.5 4.3 3.9	3–25 1–6 1–4	4 5 3
Reexploration for bleeding (%)	4.9	0–6	7
Early mortality (%)	9.0	0–13	10

**CABG** = coronary artery bypass grafting

Located after line **186**

### Early mortality

10 studies were included and the combined all-cause early mortality was 9.0% [7, 9, 12, 20–25, 33].

### Reoperation for bleeding

7 studies were included and the combined bleeding reoperation rate was 4.9% (17 patients) [12, 20–24, 33].

### Late mortality

**Fig. 2:**
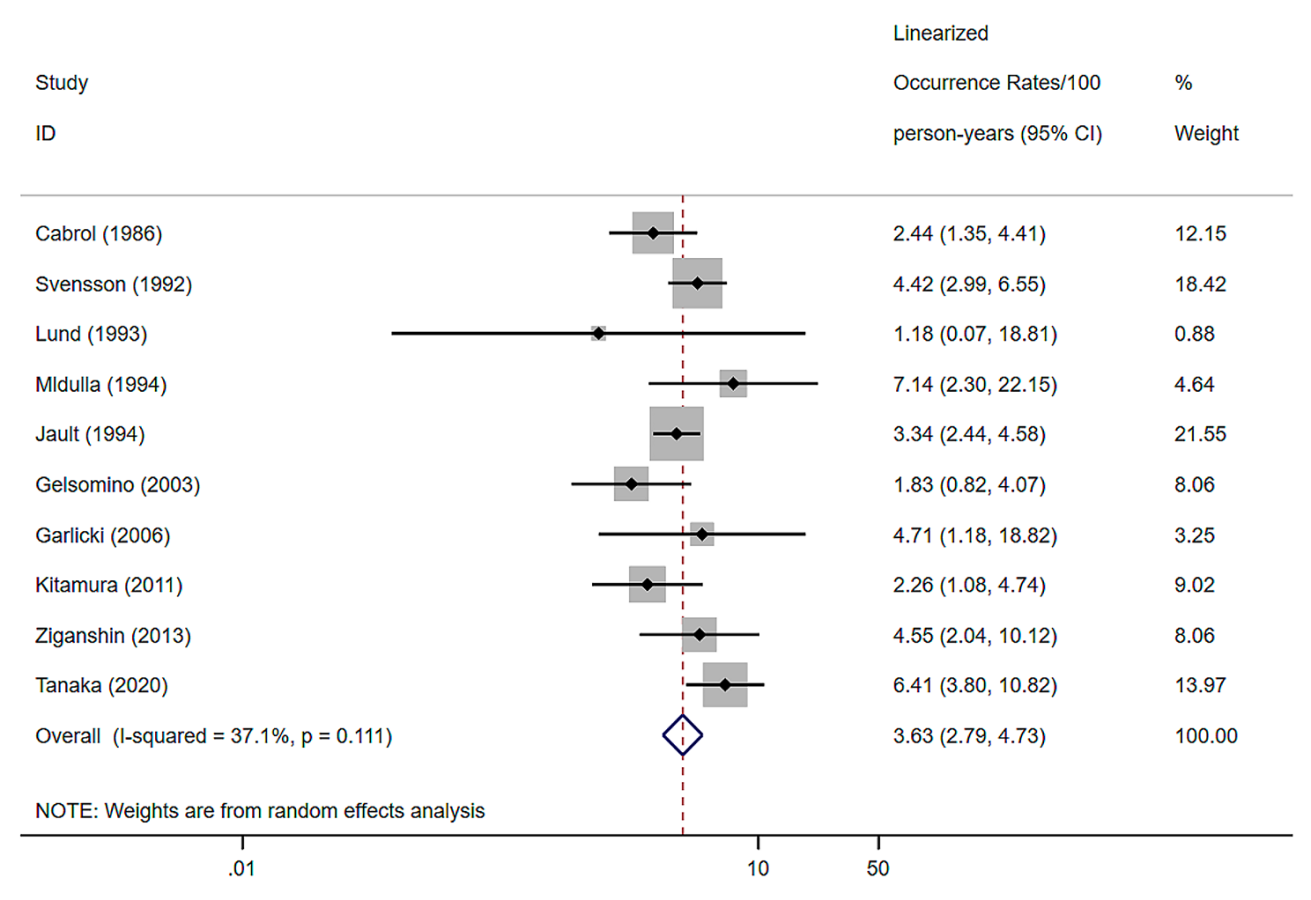
Forest plots and 95% confidence intervals for combined late mortality

10 studies were included, with a combined late mortality of 3.63% (per patient-year) using the random effects model, with a 95% confidence interval of (2.79–4.73) and a heterogeneity I^2^ of 37.1% [7, 9, 12, 20–25, 33] ( Fig. 2).

### Root reoperation

**Fig. 3:**
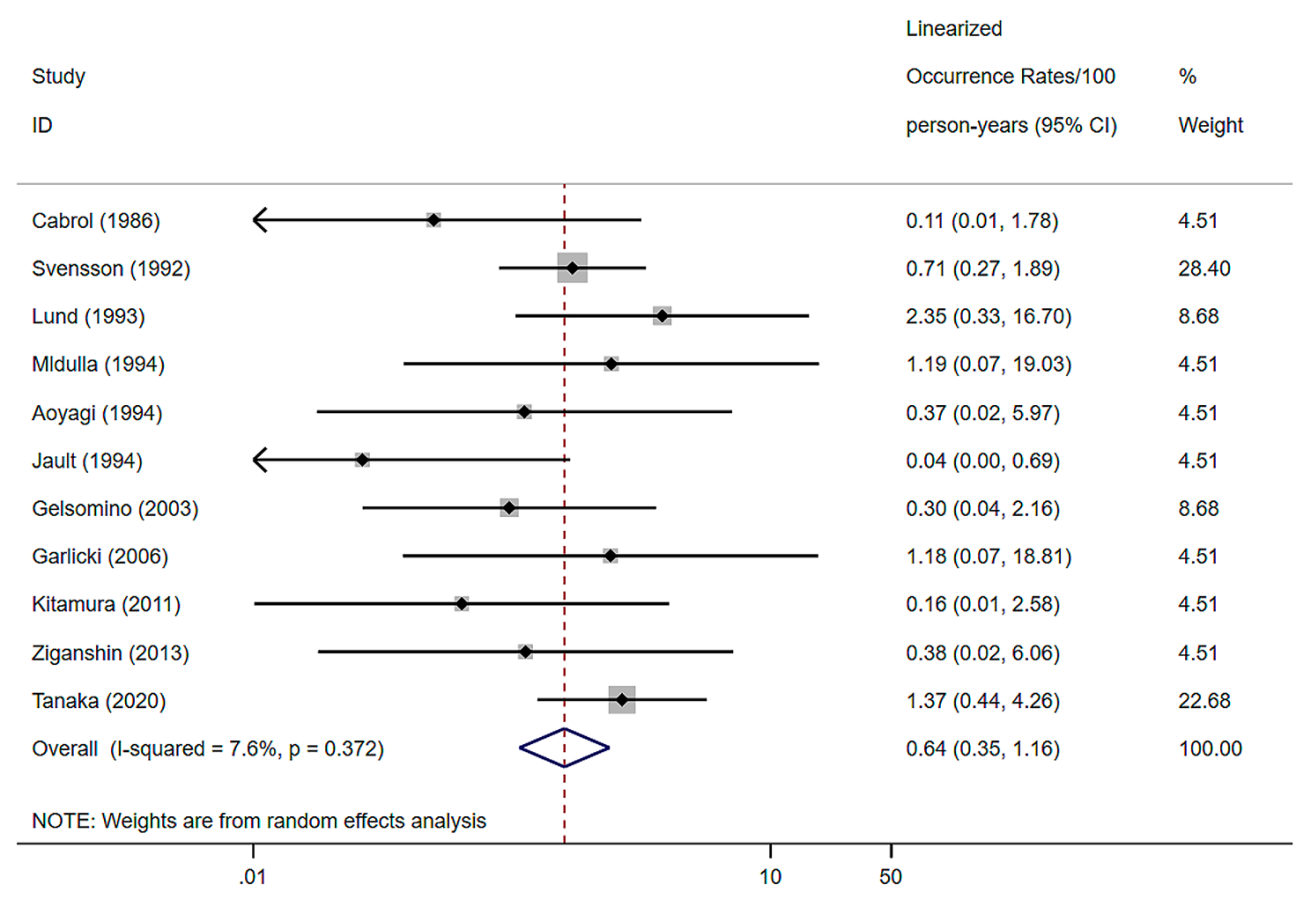
Forest plots and 95% confidence intervals for combined aortic root reoperation

The definition of aortic root reoperation followed the description of reinterventions in the guidelines for reporting mortality and morbidity after cardiac valve interventions [27]. 11 studies were included, with a random effects model combined root reoperation of 0.64% (per patient-year), a 95% confidence interval of (0.35–1.16) and a heterogeneity I^2^ of 7.6% [7–9, 12, 20–25, 33] (Fig. 3).

### Hemorrhage

**Fig. 4:**
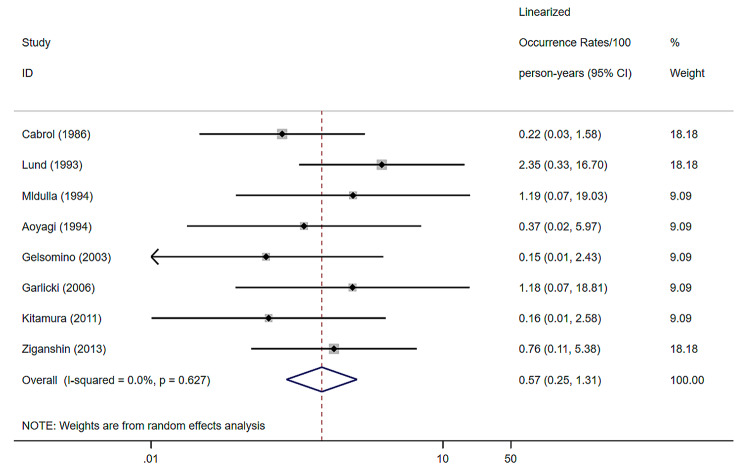
Forest plots and 95% confidence intervals for Hemorrhage

8 studies were included, with a random effects model combined hemorrhage of 0.57% (per patient-year), 95% confidence interval of (0.25–1.31) and heterogeneity I^2^ of 0% [8, 9, 12, 20–22, 24, 33] (Fig. 4).

### Thromboembolism

**Fig. 5:**
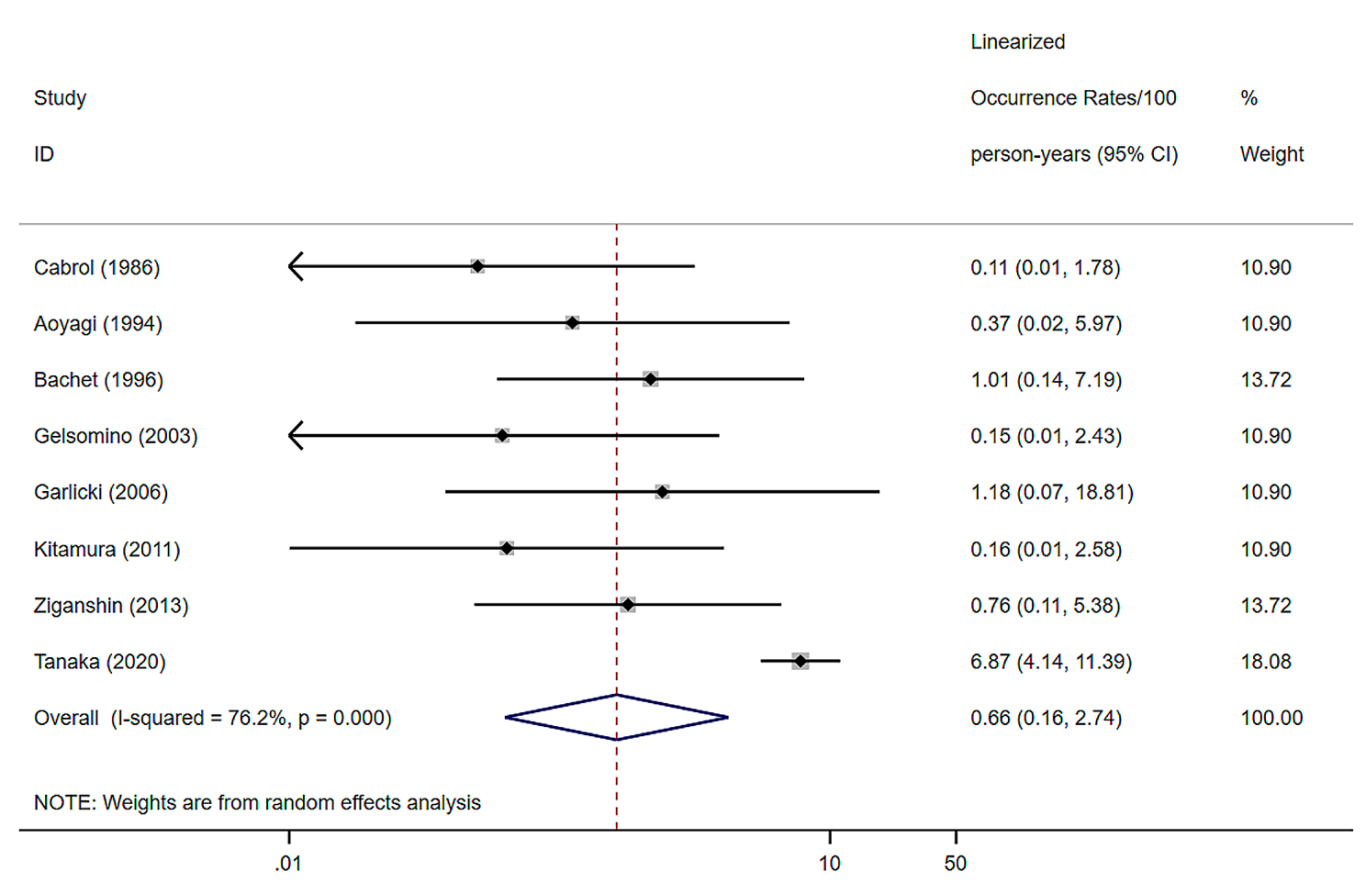
Forest plots and 95% confidence intervals for Thromboembolism

8 studies were included, with a random effects model combined thromboembolism of 0.66% (per patient-year), a 95% confidence interval of (0.16–2.74) and a heterogeneity I^2^ of 76.2% [8, 10, 12, 20–23, 33] (Fig. 5).

### Endocarditis

**Fig. 6:**
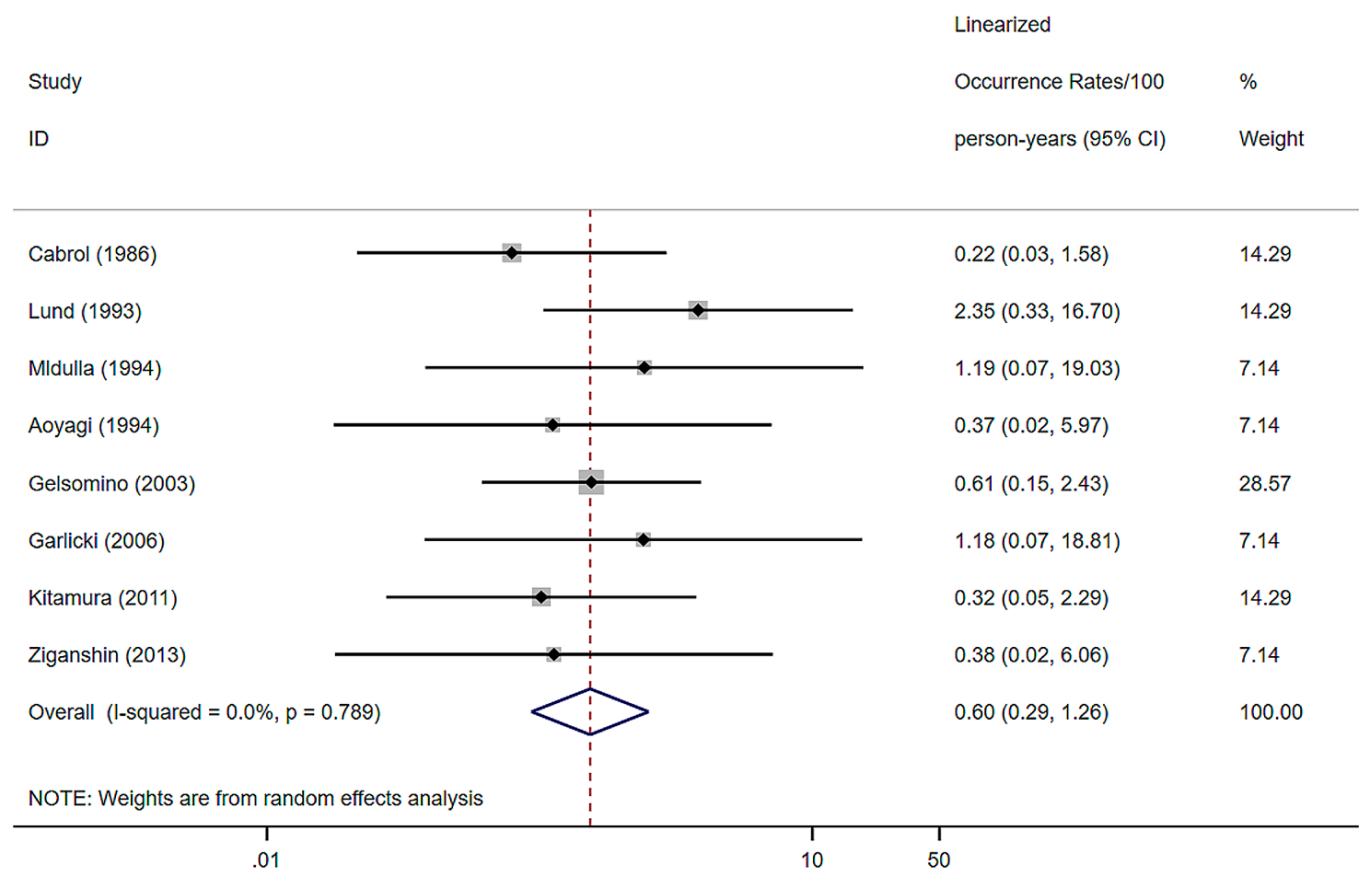
Forest plots and 95% confidence intervals for endocarditis

8 studies were included, with a random effects model for combined endocarditis of 0.60% (per patient-year), a 95% confidence interval of (0.29–1.26) and a heterogeneity I^2^ of 0% [8, 9, 12, 20–22, 24, 33] (Fig. 6).

### Major valve-related adverse events

**Fig. 7:**
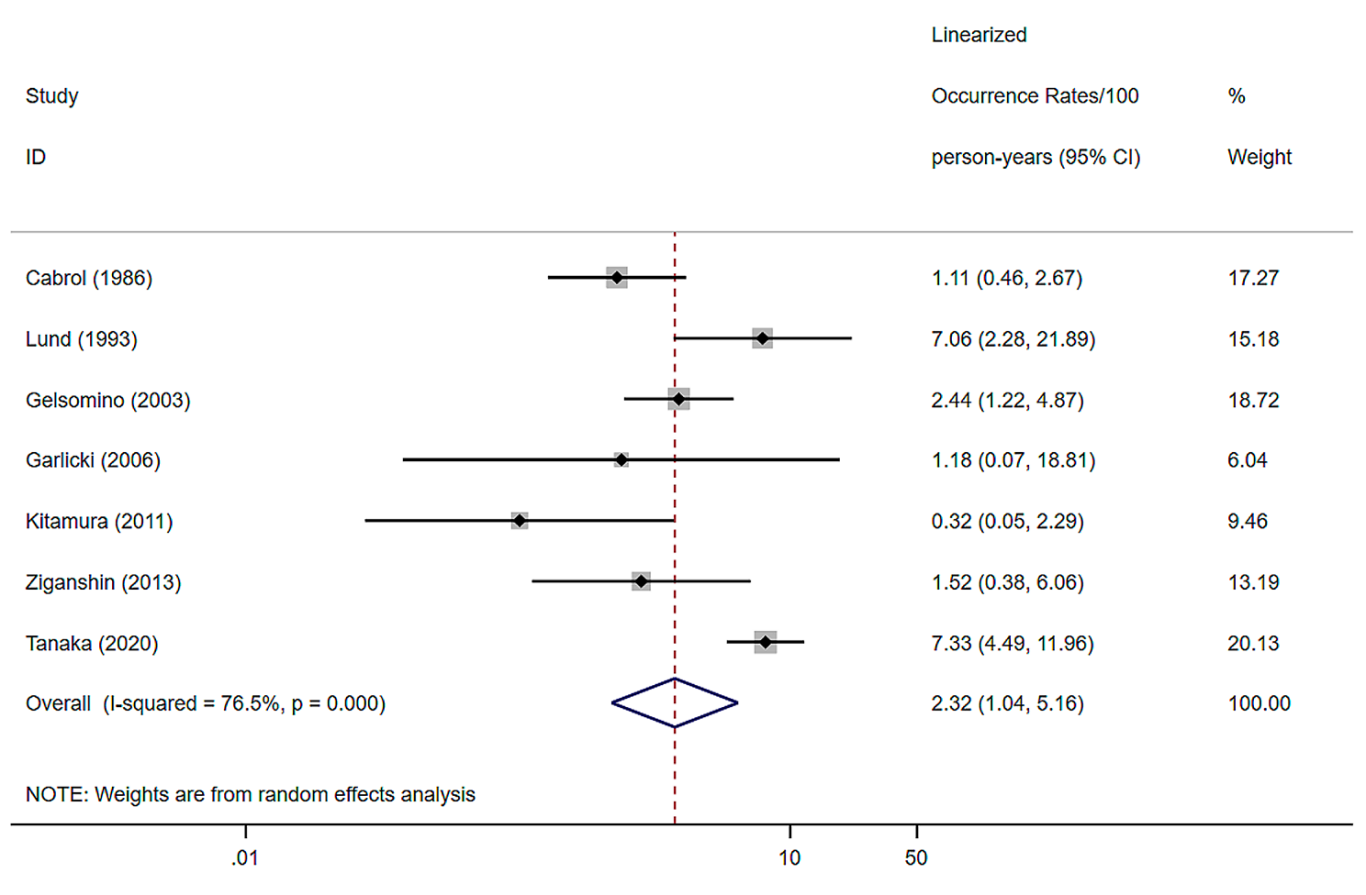
Forest plots and 95% confidence intervals for Major valve-related adverse events

7 studies were included, with a random effects model combining MAVRE of 2.32% (per patient-year), a 95% confidence interval of (1.04–5.16) and a heterogeneity I^2^ of 76.5% [9, 12, 20–23, 33] (Fig. 7).

### Cabrol-related coronary graft complications

**Fig. 8:**
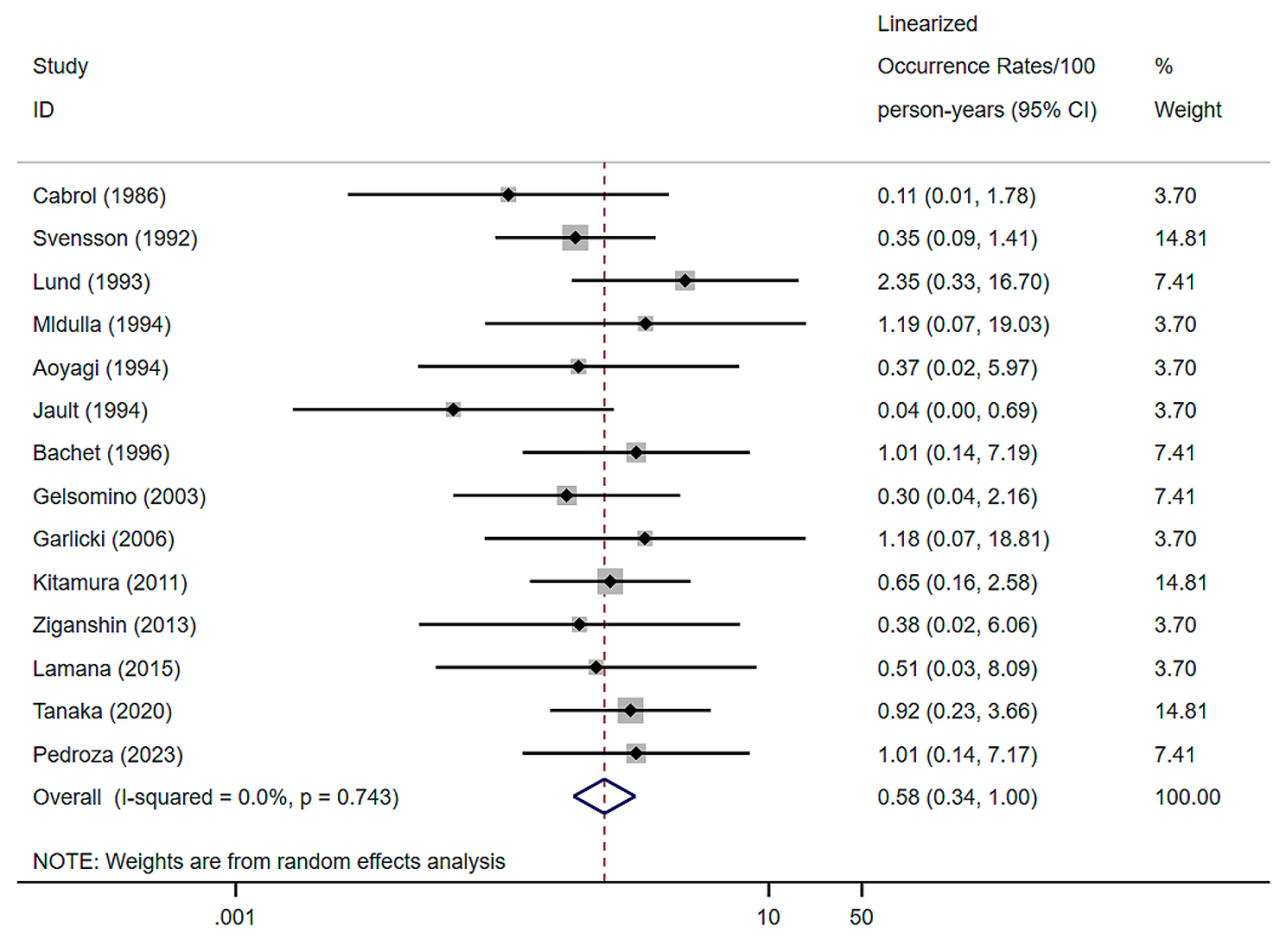
Forest plots and 95% confidence intervals for Cabrol-related coronary graft complications

14 studies were included, with a random effects model combined with a Cabrol-related coronary graft complication of 0.58% (per patient-year), a 95% confidence interval of (0.34–1.00) and a heterogeneity I^2^ of 0% [7–10, 12, 20–25, 33–35] (Fig. 8).

### Regression and sensitivity analysis

Regression analysis of baseline variables and outcome events revealed that the mode of surgery was associated with bleeding reoperation (*p* = 0.025 < 0.05), which may be a source of heterogeneity in bleeding reoperation. The remaining regression analyses did not reach statistical significance.

Sensitivity analysis was conducted on outcome events with I^2^ > 50%, and after excluding articles that may interfere with the outcome, there was no significant change in heterogeneity. A considerable heterogeneity (I^2^ > 75%) may be due to the patient’s basic condition [29], severity and complexity of the condition, as well as the surgeon’s surgical approach.

### Publication bias

Egger’s test *P* = 0.000 < 0.05 for the embolism outcome event, implying that the funnel plot was asymmetrical and therefore a publication bias could be judged for the results of the study on embolism. No publication bias was found for the remaining studies.

## Discussion

Since the inception, the Cabrol procedure has been employed and refined by numerous surgeons, the outcomes at early and late stages were also recorded [15, 16, 19, 20–23, 35]. Despite the occurrence of complications such as coronary graft thrombosis or embolism [7, 9–14], the procedure has stood the test of time and practice and has proven to be a valuable tool for surgeons in specific clinical scenarios [22, 24]. To the best of our knowledge, this study represents the most comprehensive meta-analysis of the postoperative characteristics and prognostic outcomes of Cabrol procedures published to date. It provides a significant real-world experience and a valuable reference for individual surgeons or surgical teams to select a safer and more appropriate procedure.

The combined all-cause early mortality is 9.0% and 10-year cumulative late mortality is 36.3% observed in our study which exceeds the same index of Mookhoek et al. [36] (Bentall meta-analysis) with 5.6% for early mortality and 20.2% for 10-year cumulative late mortality and Arabkhani et al. [37] (VSRR meta-analysis) with 2.2% and 15.3%, respectively. However, we have already noted that post-operative outcomes can be influenced by pre-existing conditions, such as patient selection, average patient age, comorbidities and general health. In the studies conducted by Maureira et al. [17] and Tanaka et al. [23], The rates of connective tissue disease, reoperation and aortic dissection were 13.7% and 56.0%, 4.6% and 73.8%, 24.8% and 66.7%, respectively. The early mortality were 8.5% and 15%, respectively. Additionally, 5-year survival rates were 86.3% ± 2.8 and 68% ± 6, while 10-year survival rates were 73.7% ± 4.2 and 52% ± 10. In this study, connective tissue disease, reoperation, and aortic dissection accounted for 27.7%, 34.5% and 42.2%, respectively, as detailed in (Supplementary Appendix 3). Therefore, these factors could potentially result in a suboptimal early and late mortality. It is worth acknowledging that the combined early mortality reported in our analysis may have been influenced by publication bias, selective outcome reporting, or both.

The Cabrol procedure and its modifications make haemostasis a challenge due to the increased number of anastomoses. In this study, a combined reoperation rate for bleeding was 4.9%. Although the “button” technique reduces the need for two anastomoses, it is still a time-consuming procedure due to the need to move the coronary ostia, there is also a potential risk of vascular injury and the possibility of occlusion or pseudoaneurysm formation due to tension [8, 10]. In contrast, the Cabrol technique makes it possible to visualise all bleeding sites and effectively prevents the formation of pseudoaneurysms in coronary ostia [7, 12, 20, 21, 38]. Cabrol’s innovative technique of creating a shunt fistula between the periprosthetic space and the tip of the right atrial appendage is an effective means of enhancing hemostasis [33]. This feature is a major advantage of the Cabrol procedure, especially in severe coagulation disorders [24].

In recent decades, VSRR, including the David reimplantation technique [39], the Sarsam and Yacoub reconstruction technique [40], the Florida sleeve [41], and the personalised aortic root stabilization (PEARS) [42], have gained popularity due to several advantages. VSRR preserves the native valve, eliminating the risks associated with mechanical valves (anticoagulation-related thromboembolism, bleeding) and biological valves (structural valve degeneration). It provides favourable results and improves quality of life for younger patients and those with fertility concerns who wish to avoid oral anticoagulants [43]. However, there are still limitations to the promotion and application of VSRR. In the United States, less than 15% of patients have undergone VSRR with reconstruction [2], possibly due to technical complexity, a steep learning curve and a higher reoperation rate. Benedetto et al. [44] found a fourfold increased risk of reintervention with VSRR compared to conventional CVG. Yacoub et al. [45] reported an 11% probability of reoperation within 5 and 10 years for elective surgery, and Patolla et al. [46] found a 10-year reoperation rate of 12.8% in a series of 342 patients at the Mayo Clinic. In our present study, the combined 10-year cumulative reoperation rate was 6.4%, which is an encouraging outcome.

While the risk of reoperation in CVG is comparatively lower, especially in those with longer follow-up. Due to the use of anticoagulants, patients who have undergone CVG appear to have a higher risk of bleeding and thromboembolism than those who have undergone VSRR [47, 48]. Our study reported a 10-year cumulative incidence of bleeding, embolism, endocarditis, and major valve-related adverse events of 5.7%, 6.6%, 6.0%, and 23.2%, respectively, as detailed in (Supplementary Appendix 4), which is similar to a meta-analysis of Bentall surgery by Mookhoek et al. [36] (6.4%, 7.7%, 3.9%, 26.6%). In contrast, a meta-analysis conducted by Arabkhani et al. [37] reported a lower 10-year cumulative incidence of 2.3% for hemorrhage, 4.1% for embolism, and 2.3% for endocarditis, respectively. In particularly, the complications associated with Cabrol surgery are acceptable, especially as it is often used in more complex situations. Hemorrhage and thromboembolic complications have been associated with oral anticoagulants and mechanical valve implantation. However, these issues are inherent to CVG and cannot be completely avoided. Therefore, it may be advisable to suggest tailored surgical interventions based on individual patient conditions. At the same time, the decision-making process for surgery should be well informed and collaborative between the surgeon and the patient.

The uniqueness of the Cabrol procedure lies in its innovative interposition graft, making it a preferred choice for surgeons in complex cases and reoperations [26]. Despite concerns about long-term graft patency, the combined data from our study showed a lower-than-expected 10-year cumulative incidence of coronary graft complications of 5.8%. However, potential complications may be under-reported due to publication bias or selective outcome reporting, as some patients may have passed away before being admitted to hospital. Therefore, more physiological anatomy and haemodynamic graft designs in coronary revascularisation may overcome these limitations. Researchers like Pierhler [15], Mills [16], and Kourliouros [19] have proposed their own optimizations. Meanwhile, our cardiac centre has observed no complications related to the Cabrol graft while using the modified Cabrol technique with a 3–4 mm interposition vessel [18]. In further research, we found a modified Bentall procedure described by Maureira [17] and Hirasawa [49], which is actually an innovative variant of Cabrol that targets coronary grafts. With the exception of one anastomotic pseudoaneurysm, no complications related to the Cabrol graft were reported [17]. These findings indicate that the technique is feasible, simple, reproducible, and safe. Studies evaluating the effectiveness of the Cabrol procedure and its modification using interposition grafts are summarized in (Table 2). With the development of percutaneous endovascular interventions, the treatment of Cabrol graft occlusion has evolved. Minimally invasive procedures such as balloon angioplasty and stenting have become preferred over traditional reoperation [50–53], even in complex cases [54]. It is also essential to use CT or magnetic resonance aortography in conjunction with modern transthoracic echocardiography in the early postoperative period to reduce the risk of serious complications. The importance of regular evaluation cannot be emphasised enough.

**Table 2:** Early and late results of Cabrol surgery using interposition grafts for coronary artery reimplant

Published Articles	Mean follow -up years	Total patients	Patients with Cabrol procedure	Cabrol technique type	Cabrol Fifistula %	Mortality, %			Reported complications related to the Cabrol graft
						Early	Late	Survival rate, %	
Cabrol et al., 1986 [33]	4.5	100	100	Classic	—	4	12	75 at 8 y	None
Coselli et al., 1989 [32]	—	90	90	Classic	—	9	4	—	Thrombosis of graft to LCA in1 patient
Svensson et al., 1992 [7	3.6	348	157	Classic	—	8	17^a^	76 at 3 y	Occlusion of RCA in 2 patients
Lund et al., 1993 [9]	2.5	17	17	Classic	0	41	0	100 at 30 mo	Occlusion of right limb of graft in 1 patient
Mldulla et al., 1994 [24]	2.8	140	15	Classic	—	20	38^a^	52 at 5 y	None
Aoyagi et al., 1994 [8]	6.7	66	20	Classic	0	10.6*	20.3*	71 at 10 y*	None
Jault et al., 1994 [25]	5.5	339	212^b^	Classic	100	6^b^	19.6^b^	66 at 9 y	None
Bachet et al., 1996 [10]	3.8	203	26	Classic	—	7.3*	18.4*	58 at 8 y	Thrombosis of the graft in 1 patient
Gelsomino et al., 2003 [12]	7.3	45	45	Classic	62	20	16.7	59 at 10 y	Occlusion of the graft limb to LCA in 1 patient
Garlicki et al., 2006 [20]	1.7	25	25	Classic (24%) Modified (76%)	100	0	8	—	None
Kitamura et al., 2011 [21]	8.6	36	36	Classic	0	2.8	20	73 at 10 y	Occlusion of the RCA ostium in 1 patient Stenosis of the RCA ostium in 1 patient
Ziganshin et al., 2013 [22]	3.3	40	40	Modified	—	7.5	16.2	73 at 6 y	None
Lamana et al., 2015 [34]	2.6	325	38	Classic	—	9.2*	22.8*	—	None
Tanaka et al., 2020 [23]	2.6	370	84	Modified	19	15	20^a^	52 at 10y	Pseudoaneurysm of proximal anastomosis in 1 patient;Stenosis of the coronary ostium in 1 patient
Pedroza et al., 2023 [35]	5.5	57	18	Modified	—	7.0*	—	81 at 5y*	Occlusion of the graft limb to RCA in 1 patient

RCA, right coronary artery; LCA, left coronary artery. *Presented figures are for the entire group of patients in the study. a Presented figures are derived from 5 year survival rates in the text. b Presented figures are derived from the description in the subgroup.

Located after line **349**

### Limitations

This paper systematically analyzes retrospective observational studies, a limited number of which focus on the long-term outcomes of the Cabrol procedure. Due to the retrospective nature of the studies, the observed results should be interpreted with caution. Some studies in the review didn’t adhere to guidelines for reporting mortality and morbidity after cardiac valve interventions [27]. Consequently, it was not always possible to extract reliable information on key outcome measures. Moreover, the unavailability of individual patient data prevented the use of more reliable outcome measures beyond linearised incidence. Based on the assumption of linearity, the collective linearised outcome event rates are derived from heterogeneous data sources. Caution must be exercised in interpreting the study results, as collective outcome measures may underestimate the true incidence of late morbidity and coronary graft complications after Cabrol.

## Conclusion

The distinctive features and inherent advantages of the Cabrol surgical technique highlight its significance in the management of complex scenarios, ensuring its continued presence and relevance in the field of surgery. Our study suggests that mortality, reintervention, anticoagulation, and valve-related complications of Cabrol and its modifications are not as severe as expected, even in Cabrol-related coronary graft complications. This procedure is critical to the success of complex ascending aortic surgery, and its use should not be limited by previous experience. Thus, we strongly advocate that graft design should be more closely linked to physiological anatomy and optimised hemodynamics in coronary ostial anastomosis, as this has great potential to overcome current limitations and revive widespread acceptance.

### Electronic supplementary material

Below is the link to the electronic supplementary material.

Supplementary Material 1

## Data Availability

All data generated or analysed during this study are included in this published article [and its supplementary information files].

## Abbreviations

VSRR: Valve-sparing aortic root replacement
CVG: Composite valve graft
CABG: Coronary artery bypass grafting
MAVRE: Major valve-related adverse event
RCA: Right coronary artery
LCA: Left coronary artery
