# Supplementary material for: Cabrol procedure and its modifications: a systematic review and meta-analysis
Source: J Cardiothorac Surg. 2024 Mar 26;19:153. doi: 10.1186/s13019-024-02642-w (PMC10964695; doi:10.1186/s13019-024-02642-w)
Supplement: Supplementary file 1 — Supplementary Material 1 [file 13019_2024_2642_MOESM1_ESM.docx]

**Appendix 1**

| Database | Search strategy |
| --- | --- |
| PubMed (669) | (("Cabrol"[Title/Abstract] OR "modified cabrol"[Title/Abstract] OR "aortic root replacement"[Title/Abstract] OR "composite valve graft"[Title/Abstract]) AND ("aortic root aneurysm"[Title/Abstract] OR "aneurysm aortic root"[Title/Abstract] OR "aortic root aneurysms"[Title/Abstract] OR ("aneurysm, ascending aorta"[MeSH Terms] OR ("aort*"[Title/Abstract] AND "aneurysm ascending"[Title/Abstract]) OR ("Ascending"[Title/Abstract] AND "aort*"[Title/Abstract] AND "aneurysm*"[Title/Abstract]) OR "aneurysm ascending aortic"[Title/Abstract]) OR ("dissection, ascending aorta"[MeSH Terms] OR ("aort*"[Title/Abstract] AND "dissection ascending"[Title/Abstract]) OR ("Ascending"[Title/Abstract] AND "aort*"[Title/Abstract] AND "dissection*"[Title/Abstract]) OR "dissection ascending aortic"[Title/Abstract] OR "dissection aortic root"[Title/Abstract] OR "root dissection aortic"[Title/Abstract] OR "aortic root dissection*"[Title/Abstract]) OR ("aortic valve insufficiency"[MeSH Terms] OR "insufficiency aortic valve"[Title/Abstract] OR "aortic valve incompetence"[Title/Abstract] OR "incompetence aortic valve"[Title/Abstract] OR "aortic regurgitation"[Title/Abstract] OR "regurgitation aortic"[Title/Abstract] OR "regurgitation aortic valve"[Title/Abstract] OR "aortic incompetence"[Title/Abstract] OR "incompetence aortic"[Title/Abstract]))) NOT ("case reports"[Publication Type] OR "case report"[Title]) |
| Embase (826) | ('aortic root aneurysm'/exp OR 'aortic sinus aneurysm'/exp OR 'ascending aortic aneurysm'/exp OR 'type a aortic dissection'/exp OR 'aortic regurgitation'/exp) AND ('cabrol procedure'/exp OR 'cabrol technique'/exp OR 'modified cabrol':ab,ti OR 'aortic root replacement':ab,ti OR 'composite valve graft':ab,ti) NOT 'case report':ab,ti |
| Web of Science (1025) | (((AB=(aortic root aneurysm)) OR AB=(aneurysm,ascending aorta)) OR AB=(dissection, ascending aorta)) AND ((((AB=(cabrol)) OR AB=(modified cabrol)) OR AB=(aortic root replacement)) OR AB=(composite valve graft)) NOT (TI=(case report)) |

**Appendix 2**

| **Variables** | **Description** |
| --- | --- |
| First author |  |
| Publication Year |  |
| Journal |  |
| Country | Country where patients were operated in |
| Operative period |  |
| Patient Number |  |
| Follow-up | Mean in years; calculated from median and interquartile range when mean was not given |
| Age | Mean in years; calculated from median and interquartile range when mean was not given |
| Sex |  |
| CTD |  |
| BAV |  |
| Prior cardiac surgery |  |
| Prior aortic valve surgery |  |
| Acute type A dissection | As indication for Cabrol procedure |
| Acute infective endocarditis | As indication for Cabrol procedure |
| Emergency surgery | Within 24 hours after diagnosis |
| Homograft surgery | Patients with non-Cabrol procedures |
| Valve-sparing surgery | Patients with non-Cabrol procedures |
| Other surgery | Patients with non-Cabrol procedures |
| Mechanical prosthesis | Valve choice for Cabrol procedure |
| Bioprosthesis | Valve choice for Cabrol procedure |
| Aortic hemiarch repair |  |
| Aortic arch repair |  |
| CABG |  |
| Mitral valve surgery |  |
| Reexploration for bleeding | In-hospital or within 30 days post-operatively |
| Early mortality | In-hospital mortality and 30 days mortality |
| Late mortality | According to guidelines (27) |
| Root reoperation | According to guidelines (27) |
| Hemorrhage | According to guidelines (27) |
| Thromboembolism | According to guidelines (27) |
| Endocarditis | According to guidelines (27) |
| MAVRE | Composite of late valve-related mortality, reoperation, hemorrhage, thromboembolism and endocarditis |
| Cabrol-related graft complications | Reported complications relatedto the Cabrol graft |

CTD indicates connective disease; BAV, bicuspid aortic valve; CABG, coronary artery bypass grafting; MAVRE, major adverse valve-related event

**Appendix 3**

| **First author** | **Publication**  **year** | **Country** | **Operative**  **period** | **Patients (N)** | **Mean age (years)** | **Sex**  **(% male)** | **Mean follow-up**  **(years)** | **CTD**  **(% )** | **Preoperation**  **(% )** | **AD (% )** | **Classic (% )** | **Modified (% )** |
| --- | --- | --- | --- | --- | --- | --- | --- | --- | --- | --- | --- | --- |
| Cabrol | 1986 | France | 1976-1983 | 100 | 47.5 | 84% | 4.5 | 19% | 10% | 32% | 100% |  |
| Coselli | 1989 | US | 1986-1988 | 90 | 47 | 81% |  | 24% | 26% | 41% | 100% |  |
| Svensson | 1992 | US | 1979-1991 | 157 |  |  | 3.6 |  |  |  | 100% |  |
| Lund | 1993 | Denmark | 1988-1990 | 17 | 47 | 76% | 2.5 | 18% | 18% | 47% | 100% |  |
| Mldulla | 1994 | US | 1986-1994 | 15 | 56 |  | 2.8 |  |  |  | 100% |  |
| Aoyagi | 1994 | Japan | 1973-1992 | 20 | 42.5 |  | 6.7 |  |  |  | 100% |  |
| Jault | 1994 | France | 1979-1991 | 212^b^ | 53.6 |  | 5.5 |  |  |  | 100% |  |
| Bachet | 1996 | France | 1973-1994 | 26 | 45 |  | 3.8 |  |  |  | 100% |  |
| Gelsomino | 2003 | Italy | 1986-2002 | 45 | 58.7 | 84% | 7.3 | 18% | 13% | 38% | 100% |  |
| Garlicki | 2006 | Poland | 2001-2005 | 25 | 53 | 88% | 1.7 | 8% | 28% |  | 24% | 76% |
| Kitamura | 2011 | Japan | 1988-2001 | 36 | 45 | 66% | 8.6 | 42% | 11% | 28% | 100% |  |
| Ziganshin | 2013 | US | 1995-2012 | 40 | 60 | 88% | 3.3 | 5% | 40% | 33% |  | 100% |
| Lamana | 2015 | Brazil | 2002-2013 | 38 |  |  | 2.6 |  |  |  | 100% |  |
| Tanaka | 2020 | US | 1991-2018 | 84 | 48 | 24% | 2.6 | 56% | 74% | 67% |  | 100% |
| Pedroza | 2023 | US | 2004-2020 | 18 | 53 |  | 5.5 |  | 100% |  |  | 100% |

*Presented figures are for the entire group of patients in the study. b Presented figures are derived from the subgroup.

**Appendix 4**

| **First author** | **Publication year** | **Early mortality** | **Reexploration for bleeding** | **Late mortality** | **Root reoperation** | **Hemorrhage** | **Thromboembolism** | **Endocarditis** | **MAVRE** | **Complications of grafts** |
| --- | --- | --- | --- | --- | --- | --- | --- | --- | --- | --- |
|  |  | % | % / 100-year | % / 100-year | % / 100-year | % / 100-year | % / 100-year | % / 100-year | % / 100-year | % / 100-year |
| Cabrol | 1986 | 4 | 0.89 | 2.44 | 0.11 | 0.22 | 0.11 | 0.22 | 1.11 | 0.11 |
| Coselli | 1989 | 9 |  |  |  |  |  |  |  |  |
| Svensson | 1992 | 8 |  | 4.42 | 0.71 |  |  |  |  | 0.35 |
| Lund | 1993 | 41 |  | 1.18 | 2.35 | 2.35 |  | 2.35 | 7.06 | 2.35 |
| Mldulla | 1994 | 20 | 1.19 | 7.14 | 1.19 | 1.19 |  | 1.19 |  | 1.19 |
| Aoyagi | 1994 | 10.6* |  |  | 0.37 | 0.37 | 0.37 | 0.37 |  | 0.37 |
| Jault | 1994 | 6^b^ |  | 3.34 | 0.04 |  |  |  |  | 0.04 |
| Bachet | 1996 | 7.3* |  |  |  |  | 1.01 |  |  | 1.01 |
| Gelsomino | 2003 | 20 | 0.30 | 1.83 | 0.30 | 0.15 | 0.15 | 0.61 | 2.44 | 0.30 |
| Garlicki | 2006 | 0 | 1.18 | 4.71 | 1.18 | 1.18 | 1.18 | 1.18 | 1.18 | 1.18 |
| Kitamura | 2011 | 2.8 | 0.32 | 2.26 | 0.16 | 0.16 | 0.16 | 0.32 | 0.32 | 0.65 |
| Ziganshin | 2013 | 7.5 | 4.55 | 4.55 | 0.38 | 0.76 | 0.76 | 0.38 | 1.52 | 0.38 |
| Lamana | 2015 | 9.2* |  |  |  |  |  |  |  | 0.51 |
| Tanaka | 2020 | 15 | 2.29 | 6.41 | 1.37 |  | 6.87 |  | 7.33 | 0.92 |
| Pedroza | 2023 | 7.0* |  |  |  |  |  |  |  | 1.01 |

*Presented figures are for the entire group of patients in the study. b Presented figures are derived from the subgroup.
